# The gender, age and risk factor distribution differs in self-reported allergic and non-allergic rhinitis: a cross-sectional population-based study

**DOI:** 10.1186/s13223-015-0101-1

**Published:** 2015-12-04

**Authors:** Lucia Cazzoletti, Marcello Ferrari, Mario Olivieri, Giuseppe Verlato, Leonardo Antonicelli, Roberto Bono, Lucio Casali, Isa Cerveri, Pierpaolo Marchetti, Pietro Pirina, Andrea Rossi, Simona Villani, Roberto de Marco

**Affiliations:** Unit of Epidemiology and Medical Statistics, Department of Diagnostics and Public Health, University of Verona, c/o Istituti Biologici II, Strada Le Grazie 8, 37134 Verona, Italy; Unit of Respiratory Diseases, Department of Internal Medicine, University of Verona, Verona, Italy; Unit of Occupational Medicine, University Hospital of Verona, Verona, Italy; Allergy Unit, Department of Internal Medicine, Azienda Ospedaliero-Universitaria Ospedali Riuniti, Ancona, Italy; Department of Public Health and Pediatrics, University of Turin, Turin, Italy; Unit of Respiratory Diseases, Department of Internal Medicine, University of Perugia, Perugia, Italy; Division of Respiratory Diseases, Istituto di Ricovero e Cura a Carattere Scientifico “San Matteo” Hospital Foundation, University of Pavia, Pavia, Italy; Institute of Respiratory Diseases, University of Sassari, Sassari, Italy; Unit of Biostatistics and Clinical Epidemiology, Department of Public Health, Experimental and Forensic Medicine, University of Pavia, Pavia, Italy

**Keywords:** Allergic rhinitis, Elderly subjects, Multilevel model, Non allergic rhinitis, Environmental exposures

## Abstract

**Background:**

Few population-based studies have assessed the prevalence and the risk factors of non-allergic rhinitis (NAR) in comparison to allergic rhinitis (AR). Moreover, epidemiologic data on rhinitis in the elderly subjects and in southern Europe are scarce.

**Objective:**

This study aimed at estimating the prevalence and at comparing the risk factor distribution of AR and NAR in a general population sample aged 20–84 years in Italy.

**Methods:**

A questionnaire on respiratory symptoms and risk factors was administered to random samples of the Italian population aged 20–44 (n = 10,494) 45–64 (n = 2167) and 65–84 (n = 1030) in the frame of the Gene Environment Interactions in Respiratory Diseases (GEIRD) study. Current AR and NAR were defined according to the self-reported presence of nasal allergies or of nasal symptoms without a cold or the flu.

**Results:**

NAR showed a significant descending pattern in females from 12.0 % (95 % CI 11.1, 13.1) in the 20–44 year age class, to 7.5 % (5.4, 10.3) in the 65–84 year age class (p = 0.0009), and a roughly stable pattern in males, from 10.2 % (9.3, 11.2) to 11.1 % (8.4, 13.9) (p = 0.5261). AR decreased from 26.6 % (25.7, 27.6) in 20–44 years age class to 15.6 % (13.3, 18.0) in the 65–84 years age class (p < 0.0001), without gender difference. Subjects living near industrial plants and ex- and current smokers had a higher risk of NAR. Current smokers had a lower risk and subjects living in a Mediterranean climate a higher risk of AR.

**Conclusion:**

AR and NAR are fairly distinct conditions, as they have a different age, gender and risk factor distribution.

## Background

Rhinitis is an inflammation of the nasal mucosa, which is characterized by symptoms such as excessive mucus production, congestion, sneezing paroxysm, and nasal pruritus [1]. It is a prevalent chronic disorder that impairs the quality of life and is a public cost burden due to decreased job performance [2]. Traditionally, rhinitis is divided into allergic (AR) and non-allergic (NAR) rhinitis. The first one is the most common form, and is associated with an immunoglobulin E (IgE)-mediated immune response against allergens [3]. Also NAR is frequent and its symptoms are very similar to those of AR, but the affected patients lack of evident IgE-mediated allergy [1]. It is an umbrella term for a number of heterogeneous and poorly defined nasal conditions of unknown aetiology and pathophysiology [3].

Rhinitis affects about 40 % of western populations and studies report that 25–50 % of patients with rhinitis are non-allergic [4, 5]. However, data about the prevalence, the gender distribution and risk factors of NAR are of substantially lesser quantity and quality than what has been published with regard to AR [6, 7]. Furthermore, to the best of our knowledge, few studies have been conducted on this issue in southern Europe, where climatic and environmental conditions may influence the prevalence and the risk factors of upper respiratory diseases [8].

The present study aimed at investigating the self-reported prevalence of AR and NAR in the general population aged 20–84 years in Italy and to compare the risk factor distribution in self-reported allergic and non-allergic rhinitis subjects. For these purposes the data from the Gene Environment Interaction in Respiratory Diseases (GEIRD) study were used.

## Methods

### Study design

GEIRD is a two-stage multicentre study [9]. This analysis regards the first stage, in which subjects from the general population were cross-sectionally screened for respiratory symptoms. Samples of 3000 subjects aged 20–44 years (male:female = 1:1) were randomly selected in each of the seven Italian centres: Ancona, Pavia, Salerno, Sassari, Terni, Turin and Verona. Additional random samples of about 1000 subjects aged 45–64 and 65–84 years were selected in four (Pavia, Sassari, Turin and Verona) and in two (Sassari and Verona) centres, respectively. All the eligible subjects were administered a postal questionnaire up to three times in the case of non response. A final phone interview was carried out to contact non responders (Table 1).

**Table 1:** Number (response rate, %) of subjects participating in the GEIRD study, according to centre and to their age-class at enrolment

Centre	Age class (years)		
	20–44	45–64	65–84
Ancona^a^	1866 (61.9)		
Pavia^b^	966 (37.1)	460 (54.9)	
Salerno^a^	1806 (64.7)		
Sassari^a^	1245 (53.0)	529 (62.8)	439 (44.3)
Terni^a^	1660 (59.1)		
Turin^b^	1205 (54.6)	502 (60.2)	
Verona^b^	1746 (67.7)	676 (70.1)	591 (60.1)
Total	10,494 (57.2)	2167 (62.3)	1030 (52.2)

Ancona, Salerno and Terni did not collect data on subjects in the (45–64) and (65–84) year age classes; Turin did not collect data on subjects in the (65–84) year age class

^a^Central or southern Italy

^b^Northern Italy

### Ethics, consent and permissions

Ethics approval was obtained in each centre from the appropriate ethics committee (Comitato Etico dell’Azienda Ospedaliero-Universitaria Ospedali Riuniti di Ancona; Comitato di Bioetica della Fondazione IRCCS Policlinico San Matteo di Pavia; Comitato Etico dell’Azienda Sanitaria Locale SA/2 di Salerno; Comitato di Bioetica dell’Azienda Sanitaria Locale di Sassari; Comitato Etico delle Aziende Sanitarie dell’Umbria di Perugia; Comitato Etico dell’Azienda Sanitaria Locale TO/2 di Torino; Comitato Etico per la Sperimentazione dell’Azienda Ospedaliera Istituti Ospitalieri di Verona). All participants were fully informed about all aspects of the research project and consented to complete and return the questionnaire. Those who did not want to answer the questionnaire simply did not send back the completed questionnaire. Moreover, they could ask for their names and addresses to be deleted from the enrollment list, by simply signing an appropriate box on the cover letter and returning it without costs.

### Screening questionnaire

The GEIRD Screening Questionnaire (available at the GEIRD webpage http://www.geird.org) is a modified version of the questionnaires used in previous national and international studies [10, 11]. Its aim was to investigate the presence of respiratory symptoms, asthma, rhinitis, chronic bronchitis, dyspnoea and eczema and the exposure to some environmental factors.

### Definition of AR and of NAR

The subjects who answered affirmatively to the question “Do you have any nasal allergies including hay fever?” were classified as subjects with current AR. The subjects who answered affirmatively to the question “Have you ever had a problem with sneezing, or a runny or a blocked nose when you did not have a cold or the flu?” and to the question “Do you still have this nose problem?” *and* negatively to the question “Do you have any nasal allergies including hay fever?” were classified as subjects with current NAR.

### Potential determinants of AR and NAR

The following individual-level factors were examined: gender, age class [(20–44), (45–64), (65–84)], smoking habits, educational level, residential area, presence of industrial plants near home, self-reported intensity of car and truck traffic near home, self-reported current asthma (use of any medicines for asthma or an attack of asthma in the last 12 months), self-reported eczema or skin allergy confirmed by a doctor. The centre-specific percentile rank of cumulative response [12], the type of contact (postal waves and telephone interview) and the season when the questionnaire was filled in were considered as potential design confounders.

Moreover, a centre-level variable was considered: geographical area (northern Italy: Pavia, Turin and Verona; central and southern Italy: Ancona, Salerno, Sassari and Terni).

### Statistical analysis

Multilevel analyses were performed to account for the hierarchically clustered structure of the data (subjects nested in centres) [13].

Age and gender-adjusted prevalence of AR and of NAR were obtained by applying marginal standardization to the estimates of multilevel logistic random intercept regression models, with level-1 units (subjects) nested into level-2 units (centres) and using a dummy indicator as the dependent variable (presence/absence of AR, presence/absence of NAR) and age, gender and design confounders as covariates [14].

Approximate percentile 95 % confidence intervals for the prevalence estimates were calculated with the random intercept model, using a non-parametric bootstrap percentile method with a number of replicates equal to 2000 [15]. The re-sampling scheme used to generate samples for the simulating study took the hierarchical data structure into account. As the level-2 units (centres) in the original data were clearly non-random, only level-1 units (individuals) were resampled, whereas the level-2 units were taken from the original sample [16].

To identify the factors associated with AR and NAR, two additional multilevel logistic random intercept regression models were fitted to the data, including all the potential determinants. The centre of Ancona was excluded from these analyses because the subjects’ information on their residential area, industrial plants near home and intensity of car traffic near home had not been collected. The presence of an interaction between age class and gender was assessed by means of the likelihood ratio test.

Multivariate associations of potential determinants with AR and NAR were expressed by odds ratios (ORs) and their 95 % CIs.

The intraclass correlation (ICC) was assessed to measure the proportion of the variance in the reporting of AR and NAR due to centre-level [17].

The statistical analysis was performed using STATA software, release 13.0 (Stata Corp, College Station, Tex).

## Results

### Response rate and main socio-demographic characteristics of subjects

The rate of participation in the screening stage of the survey ranged from 52.2 % in the age class 65–84 years to 62.3 % in the age class 45–64 years (Table 1).

The percentage of women was different across age groups (p < 0.001). It was 52.3 and 52.2 % in the 20–44 and 45–64 year age groups, respectively, but lower in the 65–84 year age group (42.7 %). Smoking habits were significantly different in the three age groups (p < 0.001): the percentage of current smokers decreased from 27.7 % in the 20–44 to 9.6 % in the 65–84 year age class, concomitantly the percentage of ex-smokers increased from 16.2 to 37.4 %.

### Main characteristics of subjects with AR and with NAR

The proportion of subjects reporting sinusitis was significantly greater in the subjects with NAR (38.6 %) than in the subjects with AR (33.9 %) (p = 0.002). On the contrary, the proportion of subjects reporting polyps tended to be slightly higher among subjects with AR than among subjects with NAR, though this difference was not significant (Table 2). The median age at onset of rhinitis was significantly lower in subjects with AR (16 years) than in subjects with NAR (18 years) (p = 0.001). In addition, the proportion of subjects who had used drugs for rhinitis in the previous 12 months was significantly greater in the subjects with AR (55.8 %) than in the subjects with NAR (28.6 %) (p < 0.001).

**Table 2:** Main characteristics of subjects with AR (n = 3363) and with NAR (n = 1586)

	AR	NAR	*p* value
Median age at onset (years) (IQR)	16 (10–25)	18 (10–30)	<0.001
Self-reported sinusitis (%)	33.9	38.6	0.002
Self-reported nasal polyposis (%)^a^	6.6	5.2	0.177
Use of drugs for rhinitis in the last 12 months (%)	55.8	29.7	<0.001

*IQR* interquartile range: (1st quartile–3rd quartile)

^a^Information not available for the centres of Ancona, Perugia/Terni and Sassari. The reported percentages refer to 1876 subjects with AR (of them 215 did not report whether they had nasal polyposis) and 855 subjects with NAR (of them 34 did not report whether they had nasal polyposis)

### Prevalence of AR and of NAR according to age class and gender

The crude prevalences by age and gender are reported in Table 3.

**Table 3:** Crude prevalence (%) of current AR, NAR, and of current rhinitis (either AR or NAR) by age class

	Age class (years)		
	20–44	45–64	65–84
Females			
AR	26.2	21.3	17.8
NAR	13.6	8.7	9.4
AR or NAR	38.8	30.0	26.2
Males			
AR	28.6	19.8	17.1
NAR	11.6	9.9	14.0
AR or NAR	39.2	29.7	31.1
Total			
AR	27.4	20.6	17.4
NAR	12.7	9.3	12.0
AR or NAR	39.1	29.9	29.4

**Fig. 1:**
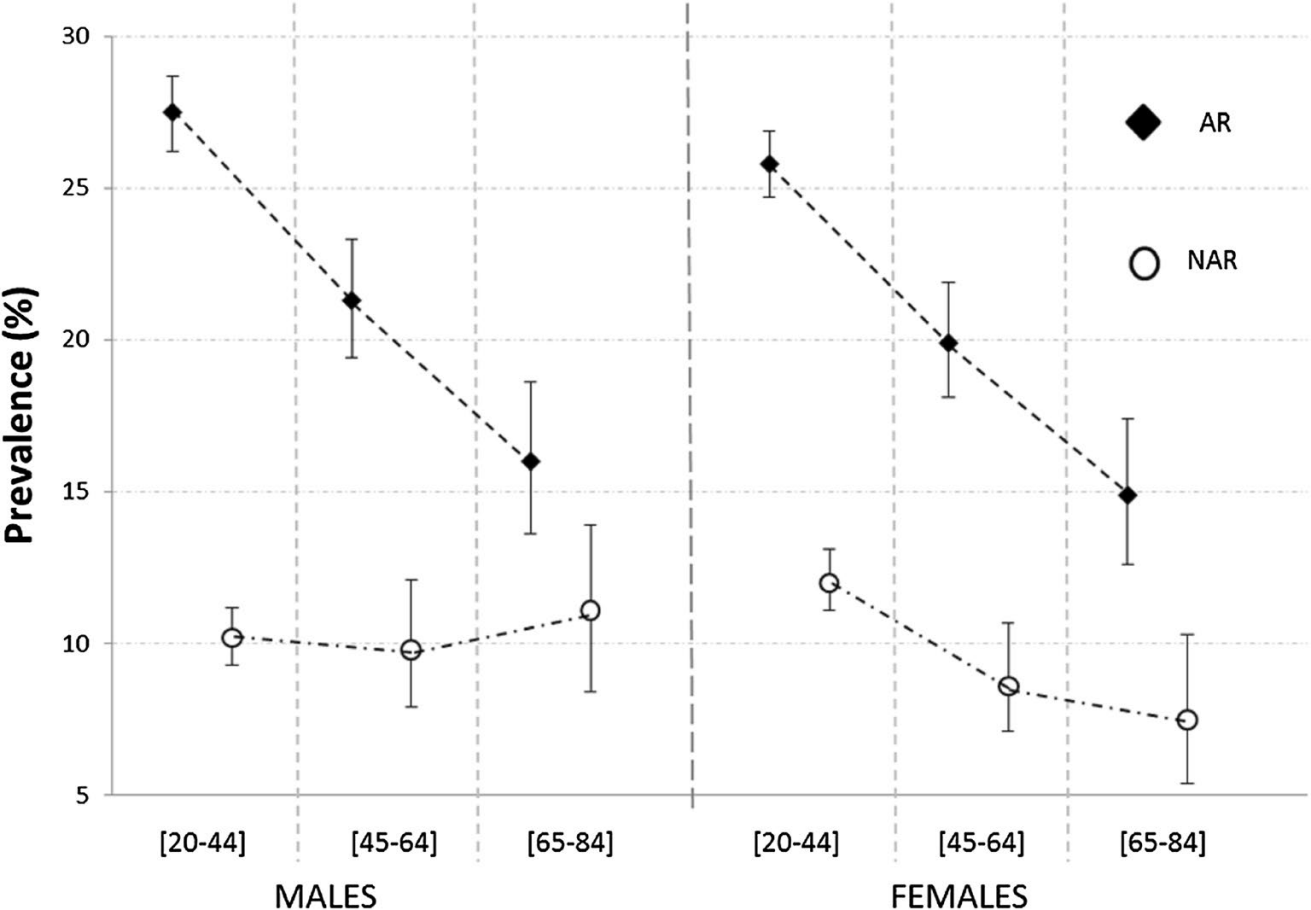
Estimated prevalence (bootstrap 95 % CI) of current AR and NAR by age class and gender, adjusted for season, percentile rank of cumulative response, and type of survey

The adjusted prevalence of AR shows a statistically significant and marked descending pattern according to age class (Table 4). The adjusted prevalence of NAR also decreases with age, but the difference among age classes is less evident and the prevalence in the 45–64 year age class is similar to that found in the 65–84 year age class. When considering the effect of gender, there was no difference between males and females with regard to the prevalence estimate of AR (Fig. 1). On the contrary, the prevalence of NAR showed a different trend according to age class in males with respect to females (Fig. 1). The prevalence of NAR showed a significant descending pattern in females from 12.0 % (95 % CI 11.1, 13.1) in the 20–44 year age class, to 7.5 % (95 % CI 5.4, 10.3) in the 65–84 year age class (p = 0.0009), and a roughly stable pattern in males (p = 0.5261), from 10.2 % (95 % CI 9.3, 11.2) to 11.1 % (95 % CI 8.4, 13.9).

**Table 4:** Estimated prevalence (%) (bootstrap 95 % CI) of current AR and NAR by age class adjusted for season, percentile rank of cumulative response, and type of survey

	Age class (years)			
	20–44	45–64	65–84	p value
AR	26.6 (25.7–27.6)	20.6 (18.8–22.5)	15.6 (13.3–18.0)	<0.0001
NAR	11.1 (10.4–11.9)	9.3 (7.9–10.6)	9.5 (7.7–11.5)	0.0418

### Determinants of AR and of NAR

After including all the potential determinants, the estimated ICC for the model describing AR was nearly equal to 0 (that is <0.0001), indicating that the centre-effect is of marginal importance in determining the reporting of AR.

Subjects living in northern Italy and current smokers were significantly less likely to report AR (Table 5). On the contrary, highly educated subjects, subjects with current asthma and subjects with eczema showed a higher risk of reporting AR. None of the other environmental exposures were significantly associated with AR.

**Table 5:** Adjusted ORs (95 % CI) for the effect of socio-demographic and environmental determinants on the reporting of AR (n = 11,606) and of NAR (n = 11,525)

	AR	NAR
Smoking habits		
Non smoker	1.00	1.00
Ex-smoker	0.90 (0.80;1.36)	*1.20 (1.02;1.41)*
Current smoker	*0.81 (0.72;0.91)*	*1.43 (1.24;1.66)*
Educational level		
Elementary school graduate	1.00	1.00
Middle school graduate	1.04 (0.80;1.36)	1.17 (0.84;1.63)
High school	*1.34 (1.04;1.73)*	1.11 (0.80;1.53)
College/university	*1.49 (1.15;1.95)*	1.01 (0.72;1.42)
Geographical area		
Central and Southern Italy	1.00	1.00
Northern Italy	*0.88 (0.80;0.97)*	0.55 (0.18;1.70)
Residential area		
City center	1.00	1.00
Suburban area	0.91 (0.81;1.01)	*0.85 (0.74;0.98)*
Rural area	0.91 (0.77;1.08)	*0.74 (0.59;0.92)*
Presence of industrial plants near home		
No	1.00	1.00
Yes	1.10 (0.98;1.25)	*1.20 (1.02;1.41)*
Intensity of car traffic near home		
Occasional/none	1.00	1.00
Frequent/constant	0.95 (0.83;1.08)	0.96 (0.81;1.13)
Intensity of truck traffic near home		
Occasional/none	1.00	1.00
Frequent/constant	1.03 (0.92;1.14)	1.10 (0.96;1.28)
Current asthma		
No	1.00	1.00
Yes	*6.85 (5.83;8.05)*	1.07 (0.85;1.35)
Doctor-diagnosed eczema		
No	1.00	1.00
Yes	*2.08 (1.84;2.34)*	1.05 (0.89;1.25)

Adjusted for all the variables included in the table plus gender, age class, centre-specific percentile rank of cumulative response, type of contact (postal waves and telephone interview) and season when the questionnaire was filled in. An interaction effect was found between gender and age class on NAR (p = 0.0021), and the model for NAR is adjusted for this interaction effect. ORs that are significantly different from 1 are reported in italics (p < 0.05)

After including all the potential determinants, the estimated ICC for the model describing NAR was 0.1281.

Subjects living near industrial plants had a significantly higher risk, while subjects living in suburban and in rural areas had a significantly lesser risk to report NAR (Table 5). Ex- and current smokers had a higher risk of reporting NAR than non-smokers. There was no association between asthma and NAR and between eczema and NAR.

Females were significantly less likely to report AR than males and subjects in the older age classes [(45–64), (65–84) years] were significantly less likely to report AR than subjects in the (20–44) year class. (Table 6). As there was a significant interaction between gender and age class (p = 0.0021), a different pattern of association between age class and NAR was estimated in males and females (Table 6): females in the (20–44) age class showed a higher risk of reporting NAR than females in the older age class whereas NAR was similarly frequent in all age classes in males [even though males in the (65–84) year age class had a non-significant higher risk of reporting NAR than males in the (20–44) age class].

**Table 6:** Adjusted ORs (95 % CI) for the effect of gender and age class on the reporting of AR (n = 11,606) and on the reporting of NAR (n = 11,525)

	AR	NAR	
Gender			
Male	1.00		
Female	*0.83 (0.75;0.91)*		

		Interaction gender × age class: p = 0.0021	
		Male	Female
Age class (years)			
20–44	1.00	1.00	*1.22 (1.06;1.70)*
45–64	*0.75 (0.65;0.87)*	0.95 (0.73;1.23)	0.85 (0.66;1.11)
65–84	*0.53 (0.42;0.68)*	1.21 (0.86;1.70)	0.64 (0.40;1.02)

Adjusted for smoking habits, educational level, geographical area, residential area, presence of industrial plants near home, intensity of car and truck traffic near home, current asthma and doctor-diagnosed eczema, plus centre-specific percentile rank of cumulative response, type of contact (postal waves and telephone interview) and season when the questionnaire was filled in. ORs that are significantly different from 1 are reported in italics (p < 0.05)

For AR, the table shows the main effect of gender and age class separately, for NAR, the table shows the ORs that describe the interaction effect between gender and age class

## Discussion

Our study is one of the few offering a view on the prevalence of AR and NAR in people aged 20–84 years. It documents that AR and NAR are major health problems, affecting overall about 39 % of subjects aged 20–44 years, 30 % of subjects aged 45–64 years and 29 % of the elderly.

### AR and NAR association with age and gender

Previous studies have found that the prevalence of AR peaks around the age of 16–24 and decreases in the subsequent years up to the age of 65–70 [18, 19]. This trend was confirmed by the present study, which also demonstrated that AR became even less prevalent in the 65–85 age class and that the descending pattern of prevalence was similar in men and women. The age related decrease in the AR prevalence may be due to the allergen specific IgE level decrease that occurs with aging in atopic individuals [20]. It has also been proposed that AR is less common in subjects over 60 years of age than in younger subjects, possibly because the allergic epidemics started quite recently [1].

The age and gender related prevalence of NAR was substantially different from that of AR, since, in men, NAR prevalence was roughly stable across age classes, whereas it was negatively associated to age in women. The dissimilar pattern seen in women may be related to the reduction in oestrogen levels with age. Nasal congestion has been reported with pregnancy, menses, menarche, and the use of oral contraceptives [21].

One interesting finding of the present study was that subjects with NAR reported the onset of symptoms at older ages compared to subjects with AR. The reason for this result is not clear. We are tempted to speculate that AR, mainly linked to atopy, appears early in life, whereas NAR, which is influenced by the environment, could occur later.

### Prevalence of AR versus NAR

Data from previous studies suggest that the ratio of AR prevalence to NAR prevalence is 3:1 [6, 22, 23]. Our data shows that the ratio changed with age: it ranged from about 2.4 in young subjects, to 1.6 in subjects who were older than 64 years.

It has been suggested that the female gender may be a risk factor for the development of NAR [24]. Our results confirmed that females have a higher risk of having NAR in the population aged between 20 and 44 years, but they also showed that the opposite is true when older age classes are considered.

### Association of AR and NAR with other respiratory diseases

As expected, AR was associated with eczema and strongly linked to bronchial asthma [25]. On the contrary, we found no relationship between asthma and NAR and between eczema and NAR. Our results are in line with those reported by Burros et al. who stated that asthma almost always has an allergic basis [26]. In contrast with our results, Leynart et al. reported an association between perennial rhinitis and asthma in nonatopic subjects [6]. The reason for this discrepancy is not clear, even though the completely different definition of non allergic rhinitis may account for the difference.

Self reported sinusitis tended to be more evident in subjects with NAR. This finding is in line with what has been described by others. In a study on the Danish adolescent and adult population, subjects with NAR were found to suffer from recurring headaches and sinusitis more frequently with respect to subjects with AR [7]. In another study, a mixed sample of Portuguese patients with allergic and non allergic rhinitis were studied in a clinical setting and the authors found that sinusitis was more frequent in NAR than in AR subjects [27].

In our study, nasal polyps were more frequently reported by subjects with AR than by subjects with NAR. However, this difference was not statistically significant, and no definite conclusion could be reached.

We did not collect information on the severity of rhinitis in our study. However, the fact that subjects with AR used drugs for their nasal problem more frequently than subjects with NAR, suggests that nasal symptoms are more frequent or severe in AR. On the other hand, it is not possible to exclude that subjects with AR, belonging to the higher socio-economic class (as indirectly indicated by a higher level of education), might pay more attention to their symptoms in comparison to subjects with NAR.

### Environmental exposures

#### Smoking habits

Population studies on the association between tobacco smoking and AR have provided inconsistent results, but a recent systematic review in adults found that active smoking was associated with a decreased risk of AR [28]. Our results are in agreement with this finding as we found that current smokers were significantly less likely to report AR with respect to non-smokers.

There are few population-based studies on the association between smoking and NAR, which have consistently found an increased risk of having the disease in smokers compared to non-smokers [29–31]. Our results are in line with these studies.

The apparent protective effect of cigarette smoking on AR might be due to the fact that AR is strongly associated to asthma (Table 5), and therefore the affected subjects tend to avoid the irritant effect of smoking on their airways. As far as the positive association between smoking and NAR is concerned, we speculate that nasal mucosa may be exposed to a part of the side stream smoke which originates from the tip of the cigarette. Moreover, a proportion of smokers exhale smoke through the nose.

#### Traffic and industrial related air pollution

Several studies have established an association between increased air pollution and the increased risk of allergic sensitization and the prevalence of rhinitis worldwide [29], whereas other studies have failed to detect an association between traffic-related pollution and allergic sensitization or hay fever [30, 31].

We found no significant association of car or truck traffic, neither with AR nor with NAR. A self-reported assessment of traffic-related air pollution exposures was considered in this study, and it is possible that the lack of association between traffic frequency and reported symptoms is due to bias. However, a previous study carried out in Italy found that self-reported traffic density in residential areas was clearly associated with measured nitrogen dioxide level [32].

The presence of industrial plants near the residence was associated with a higher risk of reporting NAR, but not of reporting AR. This finding seems to indicate that some environmental exposures may play a role in the development of non-allergic nasal symptoms and diseases, but not of AR.

#### Residence in rural areas

Previous studies have found that the prevalence of AR is higher in urban than in rural areas [33]. In the current study, living in rural areas was negatively, though not significantly, associated with AR, but it was negatively and significantly associated with NAR. Up until now, little is known about how NAR correlates to urban/rural living. Nevertheless, our results are consistent with previous studies that found a trend of increasing prevalence of chronic nasal symptoms with increasing degrees of urbanization [34, 35].

#### Northern versus southern Italy

Previous research has shown that climatic factors may influence the prevalence of allergic diseases [36–38]. In the current study, subjects living in northern Italy were found to report AR less frequently than subjects living in central and southern Italy, but there was no evidence that the geographical area was associated with NAR. To our knowledge, no other studies have tried to describe the geographical distribution of NAR in Italy.

The risk of having AR was higher in central/southern Italy, which has a milder, Mediterranean climate, with respect to northern Italy, which has a sub-continental climate [39]. These differences in the prevalence of AR could be linked to variations in the climate itself or to variations in the characteristics and the length of the pollen season. On the other hand, other factors related to geo-climatic variations, such as different lifestyles, time spent outdoors and diet, may contribute to the regional differences in the prevalence of AR. The geographical variation found in the prevalence of AR, but not of NAR, is probably due to factors related to the concentration of allergens. Higher temperatures and the coastal climate (three of the seven centres that participated in the study are central/southern coastal cities) are associated with higher levels of allergen exposure [40].

#### Strengths and limits of the study

The strength of the present study is that it is a large-scale, population-based survey, which made it possible to control for confounding socio-demographic and environmental factors. A possible limitation is that it was only based on a screening questionnaire, and we performed no allergy skin tests or IgE measurements. Accordingly, the classification of rhinitis as AR or NAR was carried out using a subjective response. However, this response seems to be very specific for AR [19, 41]. In a previous survey conducted in Italy by our group, in which the same question we used to identify subjects with allergic rhinitis (‘Do you have nasal allergies, including hay fever?’) was adopted, it was found that atopy was present in most of the subjects reporting allergic rhinitis (124/157 = 79 %), whereas it was present only in a minority of the remaining subjects [19].

Data on the specificity and sensibility of the definition of NAR are lacking. It could be hypothesized that, in view of the fact that we had no skin prick test results, some of the subjects classified as affected by NAR are in reality atopic and consequently they were incorrectly categorized. However, if this speculation is true, it is likely that the differences in the distribution of the risk factors of NAR and AR would be less evident. On the contrary, a correct classification could make the differences we found even stronger.

Another limitation was the cross-sectional study design, which made it difficult to assess if some potential risk factors, such as air pollution, are associated with the onset of nasal diseases or if they only trigger symptoms of an existing illness.

## Conclusion

In conclusion, our survey indicates that AR and NAR are fairly distinct conditions. Besides having a different age and gender distribution, they also have a dissimilar age at onset. Of further interest is the fact that the two nasal diseases are related to different risk factors. NAR is associated with high pollution linked to industrial plants and smoking habits. Subjects with a higher education and current smokers had a lower risk of AR, which was more frequently reported by subjects living in a Mediterranean climate.
